# Supplementary material for: Identification of photoperiod-induced specific miRNAs in the adrenal glands of Sunite sheep (Ovis aries)
Source: Front Vet Sci. 2022 Jul 22;9:888207. doi: 10.3389/fvets.2022.888207 (PMC9354845; doi:10.3389/fvets.2022.888207)
Supplement: Supplementary file 1 [file Data_Sheet_1.ZIP › Supplementary file/miRDeep2 file.zip!/miRDeep2 file/Know_Structure/SP42A3/oar-mir-433.pdf]

miRBase precursor : oar-mir-433
Total read count : 628
oar-miR-433-5p read count : 1
oar-miR-433-3p read count : 627
remaining reads : 0

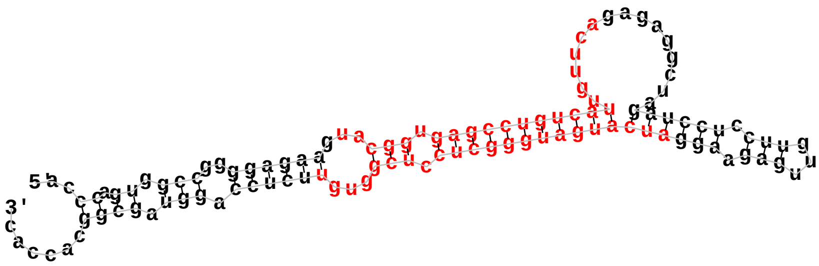

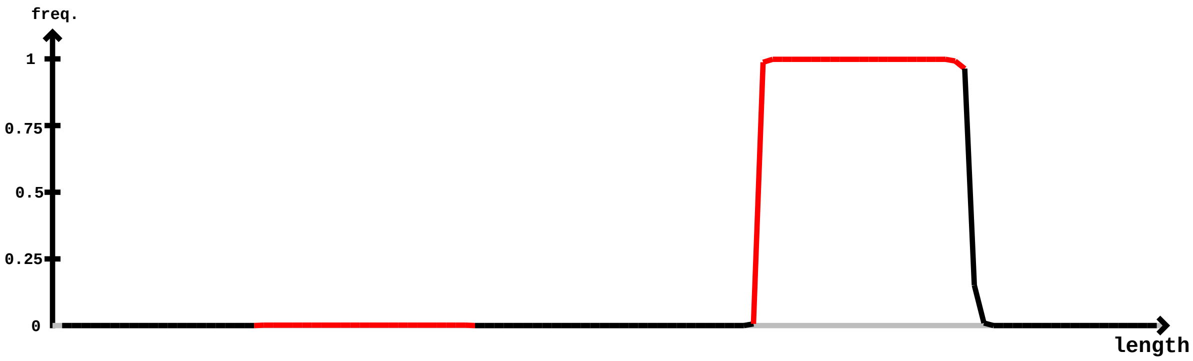

oar-miR-433-5p

oar-miR-433-3p

```
5'- acccaguggccggggagaaguacggugagccugucauuguucagagaggcuagauccuccuuguugagaaggaucaugaugggcuccucgguguucuccagguagcggcaccac   -3'   exp
    ..((.((.(((..((((((...(((.(((((((((((..............((((((.(((...))).))))))))))))))))).)))...)))))).))).)))).......   reads   mm   sample
    ....................uacggugagccugucauuguuc........................................................................   1   0   seq
    .......................................................................gaucaugaugggcuccucggugu....................   3   0   seq
    .......................................................................gaucaugaugggcuccucgguguu...................   1   0   seq
    ........................................................................aucaugaugggcuccucggu......................   4   0   seq
    ........................................................................aucaugaugggcuccucggug.....................   18   0   seq
    ........................................................................aucaugaugggcuccucggugu....................   500   0   seq
    ........................................................................aucaugaugggcuccucgguguu...................   88   0   seq
    ........................................................................aucaugaugggcuccucgguguuc..................   6   0   seq
    .........................................................................ucaugaugggcuccucggugu....................   7   0   seq
```
